# Supplementary material for: Mitochondrial Porin Is Involved in Development, Virulence, and Autophagy in Fusarium graminearum
Source: J Fungi (Basel). 2022 Sep 4;8(9):936. doi: 10.3390/jof8090936 (PMC9506537; doi:10.3390/jof8090936)
Supplement: Supplementary file 1 [file jof-08-00936-s001.zip › jof-1886744-supplementary.pdf]

## Supplementary Materials:

**Table S1** Primers used in this study.

| Primer | Sequence (5′-3′) | Application |
|---|---|---|
| porin-AF | AGTGCGAGTGTCGGTGAATG | amplify *Fgporin* 5′ flank |
| porin-AR | TTGACCTCCACTAGCTCCAGCCAAGCC GGACGGGAGAGAGAAACGG | sequence |
| porin-BF | GAATAGAGTAGATGCCGACCGCGGGTT TCCCGTTGATGTGTTCGC | amplify *Fgporin* 3′ flank sequence |
| porin-BR | CCTGGATGATTGGGTGCTT | |
| HYG-F | GGCTTGGCTGGAGCTAGTGGAGGTCAA | amplify *Hph* N-terminal |
| HY-R | AACCCGCGGTCGGCATCTACTCTATTC | sequence |
| YG-F | GATGTAGGAGGGCGTGGATATGTCCT | amplify *Hph* C-terminal |
| HYG-R | GTATTGACCGATTCCTTGCGGTCCGAA | sequence |
| porin-K1F | GAGGGTGTTGAGGTTGTGCTC | for identification of |
| porin-K1R | GTTGGCGACCTCGTATTGG | *Fgporin* deletion transformants |
| porin-K2F | GCGAAGAATCTCGTGCTTTC | for identification of |
| porin-K2R | CGCCGCTCTTACTACGCT | *Fgporin* deletion transformants |
| porin-MF | AGATACCAAACACCGCAATCA | for identification of |
| porin-MR | CTTCACGGACATAAAGAGGCA | *Fgporin* deletion transformants |
| GAPDH-F | CTTACTGCCTCCACCAACTG | quantitative RT-PCR analysis |
| GAPDH-R | TGACGTTGGAAGGAGCGAAG | |
| Tri5-F | GAGTGTTTCATGCATGGCTACGTC | quantitative RT-PCR analysis |
| Tri5-R | CTGAGCCTCCTTCACATCGTCC | |
| Tri6-F | CTGAGGGCATTCTGAGTAGCGACA | quantitative RT-PCR analysis |
| Tri6-R | CGTTATGTTTATCGGCACTTTG | |
| Tri10-F | GCGACAGGAGCAAGAACATAA | quantitative RT-PCR analysis |
| Tri10-R | GGCGGCGTAAATCTGAGTG | |
| porin-CF | CACCATCACCATCACTCGAGCAGTTCTC TGATGGTGTCGG | *Fgporin* complementation |
| porin-CR | CGCCCTTGCTCACCTCGAGTGAAAGCA TGAAGGCAACCT | |
| Tri1-GFP-F | TCGTGGTTCTCATCACCATCACCATCAC TCGAGGAAAGAGGCTGGTAGCTGCG | For *FgTri1-GFP* fusion construct generation |
| Tri1-GFP-R | CCGGTGAACAGCTCCTCGCCCTTGCTCA CGTCATCCTGTACCAATTCCAATCGC | |
| Tri4-GFP-F | TCGTGGTTCTCATCACCATCACCATCAC TCGAGAGTAGCGACAGAAAAAGCGCTT | For *FgTri4-GFP* fusion construct generation |

| | | |
|---|---|---|
| | G | |
| Tri4-GFP-R | CCGGTGAACAGCTCCTCGCCCTTGCTCACCAAAGCCTTGAGAACCTTGACTCG | |
| GFP-Atg8-1F | CGACTCACTATAGGGCGAATTGGGTACTCAAATTGGGAACAACTGAGAACTCGGGTGA | amplify *GFP-FgAtg8* sequence |
| GFP-Atg8-1R | GGTGAACAGCTCCTCGCCCTTGCTCACCATGTTGACGGTGATGGTTGTTG | |
| GFP-Atg8-2F | GGTGAACAGCTCCTCGCCCTTGCTCACCATGTTGACGGTGATGGTTGTTG | amplify *GFP-FgAtg8* sequence |
| GFP-Atg8-2R | ACCATCACCGTCAACATGGTGAGCAAGGGCGAGGAGCTGTT | |
| GFP-Atg8-3F | CCTTGAATTTGCTGCGCTTGTACAGCTCGTCCATGCCGAGAG | amplify *GFP-FgAtg8* sequence |
| GFP-Atg8-3R | CTCGGCATGGACGAGCTGTACAAGCGCAGCAAATTCAAGGACGA | |
| AD-porin-F | AGATTACGCTCATATGGCCATGGAGGCCAGTGAATTCATGTCTGTCCCCGCCTTCT | amplify pGADT7-*Fgporin* sequence |
| AD-porin-R | GATGGATCCCGTATCGATGCCCACCCGGGTGGAATTCTTAACCCTCGAAGGTGAAGC | |
| AD-Mdm35-F | TACGCTCATATGGCCATGGAGGCCAGTGAATTCATGTCAGCTTCACTATCACC | amplify pGADT7-*FgMdm35* sequence |
| AD-Mdm35-R | CGCTGCAGGTCGACGGATCCCCGGGAATTCCTACTCACGGGCCTCTTGCACCAG | |
| AD-Ups1-F | AGATTACGCTCATATGGCCATGGAGGCCAGTGAATTCATGGTTCTCACTCGCACGA | amplify pGADT7-*FgUps1* sequence |
| AD-Ups1-R | ATGGATCCCGTATCGATGCCCACCCGGGTGGAATTCTTAAGCAAGTTGACGCTGACG | |
| AD-Ups2-F | TACGCTCATATGGCCATGGAGGCCAGTGAATTCATGAAGGTCTTTAGCAACAGCG | amplify pGADT7-*FgUps2* sequence |
| AD-Ups2-R | ATGGATCCCGTATCGATGCCCACCCGGGTGGAATTCTTATAGAACCTGGCGAGCCCT | |
| BD-porin-F | TCAGAGGAGGACCTGCATATGGCCATGGAGGCCGAATTCATGTCTGTCCCCGCCTTC | amplify pGBKT7-*Fgporin* sequence |
| BD-porin-R | CCGCTGCAGGTCGACGGATCCCCGGGAATTCTTAACCCTCGAAGGTGAAGCTG | |
| porin-$YFP^N$ -F | TGGTTCTCATCACCATCACCATCACTCG | amplify *Fgporin*-$YFP^N$ |

| | | |
|---|---|---|
| | AGACTGGTACCGATGCATGTTT | sequence |
| porin-YFP$^{N}$- R | GCCCTTGCTCACCATCGTGGCGATGGA GCGACCCTCGAAGGTGAAGCTGG | |
| Ups1-YFP$^{N}$- F | TGGTTCTCATCACCATCACCATCACTCG AGAGTGTTGTGCATTACACATC | amplify *FgUps1-YFP*$^{N}$ sequence |
| Ups1-YFP$^{N}$- R | GCCCTTGCTCACCATCGTGGCGATGGA GCGAGCAAGTTGACGCTGACGTT | |
| Ups2-YFP$^{N}$- F | TGGTTCTCATCACCATCACCATCACTCG AGGTATCCGCAACCTCACAGGA | amplify *FgUps2-YFP*$^{N}$ sequence |
| Ups2-YFP$^{N}$- R | GCCCTTGCTCACCATCGTGGCGATGGA GCGTAGAACCTGGCGAGCCCTCT | |
| porin-YFP$^{C}$- F | TGGTTCTCATCACCATCACCATCACTCG AGACTGGTACCGATGCATGTTT | amplify *Fgporin-YFP*$^{C}$ sequence |
| porin-YFP$^{C}$- R | CAGGTCGTTCGGGATCTTGCAGGCCGG GCGACCCTCGAAGGTGAAGCTGG | |

**Table S2** The amino acid similarity of Fgporin with other fungal porins.

| | *Fusarium graminearum* porin | *Fusarium oxysporum* porin | *Neurospora crassa* porin | *Saccharomyces cerevisiae* porin1 | *Saccharomyces cerevisiae* porin2 |
|---|---|---|---|---|---|
| Number of Amino acids | 283 | 283 | 283 | 283 | 281 |
| Similarity | | 100% | 82% | 43% | 33% |

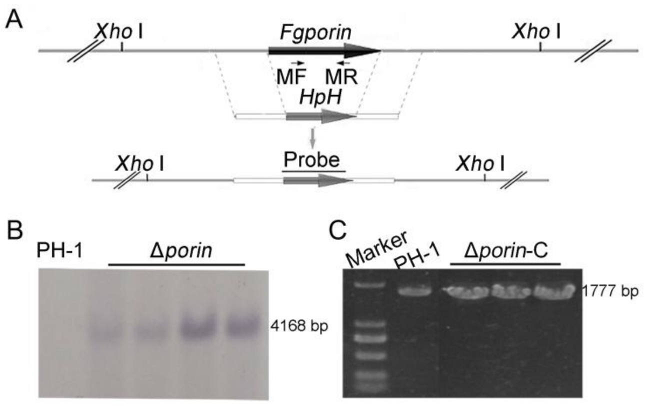

**Figure S1** Generation and identification of *Fgporin* deletion mutants. (A) Deletion strategy used for *Fgporin*. The gene replacement cassette contained the whole *hygromycin-phosphotransferase* (*HpH*) gene. (B) Southern blot analysis of *Fgporin* deletion mutants. Genomic DNA was digested with *Xho* I. (C) PCR verification of *Fgporin* complementation strains.

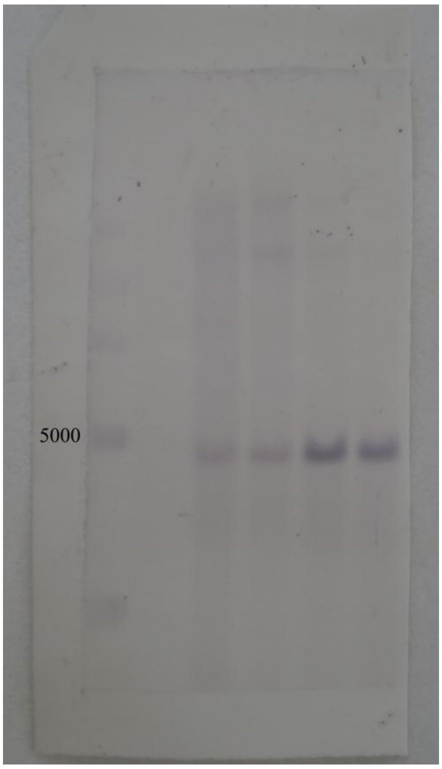


**Figure S2** Southern blot analysis of *Fgporin* deletion mutants. Genomic DNA was digested with *Xho* I.

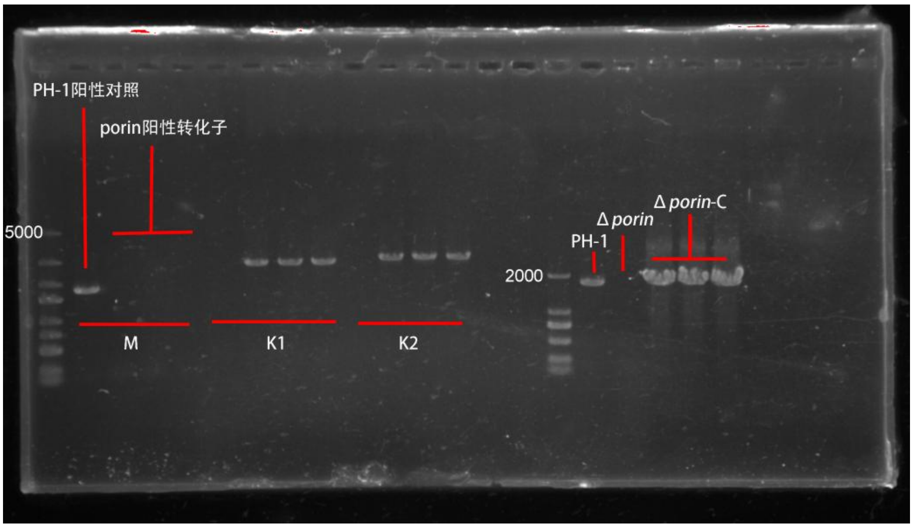


Figure S3 PCR verification of *Fgporin* deletion mutants and *Fgporin* complementation strains.

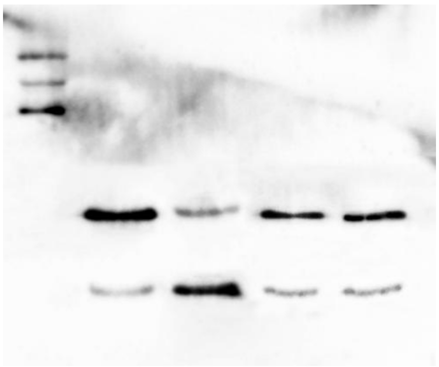

Figure S4 The original picture of Western Blot. The upper band was GFP-FgAtg8, and the lower band was GFP.

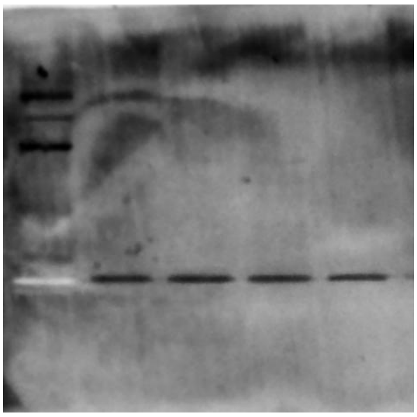

Figure S5 The original picture of Western Blot. The band was GAPDH.
